# Real‐life second‐line epirubicin–paclitaxel regimen as treatment of relapsed small‐cell lung cancer: EpiTax study

**DOI:** 10.1002/cam4.5143

**Published:** 2022-08-24

**Authors:** Josselin Annic, Hélène Babey, Romain Corre, Renaud Descourt, Gilles Quéré, Emmanuelle Renaud, Mickaël Lambert, Pierre Le Noac'h, Estelle Dhamelincourt, Jessica Nguyen, Alicia Vu, Vincent Bourbonne, Gilles Robinet, Margaux Geier

**Affiliations:** ^1^ Department of Medical Oncology, CHRU Morvan University Hospital Brest France; ^2^ Department of Pulmonary Diseases CH Cornouaille Quimper France; ^3^ Department of Radiation Oncology University Hospital Brest France; ^4^ LaTIM UMR 1101 INSERM University Brest Brest France

**Keywords:** cerebral efficacy, epirubicin, paclitaxel, real life, small‐cell lung cancer

## Abstract

**Background:**

Few therapeutic options are approved as second‐line treatment after failure of platinum‐based chemotherapy for patients with extensive‐stage small‐cell lung cancer (ES‐SCLC). Topotecan widespread use remains challenged by the risk of severe toxicities in a pretreated population. Little is known about the efficacy and safety of epirubicin–paclitaxel doublet in second‐line and beyond and especially cerebral outcomes.

**Methods:**

EpiTax is a retrospective multicenter observational real‐life study. We evaluated the efficacy of epirubicin 90 mg/m^2^ combined with paclitaxel 175 mg/m^2^ every 3 weeks in SCLC patients after failure of at least one line of platinum‐based chemotherapy. The primary endpoint was progression‐free survival (PFS). Secondary endpoints were overall survival (OS), objective response rate (ORR), disease control rate (DCR), intracranial control rate (ICR), and safety.

**Results:**

A total of 29 patients were included. The median of previous systemic therapy lines was 2 (1–4). Eleven patients received the treatment in the second line. Characteristics of patients were a median age of 60 years (45–77), 65.5% of males with 72.4% of PS 0–1. Fifteen patients had a history of brain metastases. Median PFS and OS achieved 11.0 (95% CI, 8.1–16.3) and 23 (95% CI, 14.1–29.6) weeks, respectively. ORR was 34.5% and DCR was 55.2%. ICR was 3/15 (20%). Grade 3–4 adverse events were mainly hematological and concerned 7 patients. No case of febrile neutropenia or toxic death was reported.

**Conclusion:**

Epirubicin–paclitaxel association highlighted promising efficacy with PFS and OS of 11 and 23 weeks, respectively, ORR of 34.5%, and a tolerable safety profile. This doublet could represent another valuable therapeutic option for ES‐SCLC patients treated in the second line and beyond.

## Graphical abstract

In this real‐life study, we investigate the efficacy and safety of second‐line epirubicin‐paclitaxel regimen in 29 extensive‐stage small‐cell lung cancer (ES‐SCLC) patients. Our results highlight promising survival outcomes, significative systemic activity and favorable toxicity profile. This association seemed to be effective and could represent another valuable therapeutic option in a pre‐treated ES‐SCLC population.

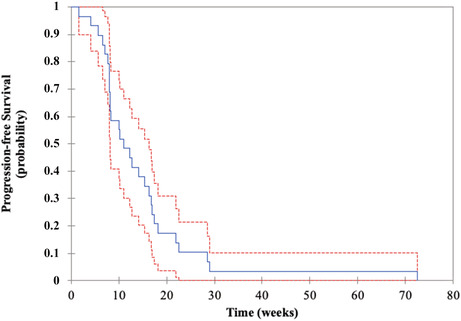

## 1 INTRODUCTION

Small‐cell lung carcinomas (SCLC) represent about 15% of lung cancers. The decrease in SCLC incidence over time is explained by a decline in consumption of tobacco. Despite initial chemosensitivity of most SCLC, relapse is almost systematic and characterized by a metastatic aggressive profile. Extensive‐stage (ES) SCLC accounts for 60–70% of cases. Brain metastases (BMs) concerned 10% of patients at initial diagnosis and up to 50% during the course of the disease. During the past two decades, standard metastatic first‐line treatment consisted of platinum plus etoposide association,^1^, ^2^ with an objective response rate (ORR) of 60%, a median progression‐free survival (PFS), and overall survival (OS) of 4 and 8 months, respectively. Recently, the addition of immunotherapy to standard first‐line chemotherapy has added a modest but significant improvement in survival outcomes.^3^, ^4^

Few therapeutic options exist, however, as second‐line treatment. Rechallenge with platinum‐etoposide regimen has been compared with oral topotecan and offers a reasonable option for patients with sensitive relapsed SCLC.^5^, ^6^ Topotecan, a topoisomerase I inhibitor, is formally approved in a second‐line setting for relapsed ES‐SCLC, based on the improvement of patient outcomes versus best supportive care alone (median OS of 25.9 weeks, ORR of 7%).^7^, ^8^ Nevertheless, severe hematological toxicities often occur leading to a challenging prescription. Topotecan has also been compared with other chemotherapies such as cyclophosphamide, adriamycin, and vincristine combination (CAV), but also monotherapy regimens such as vinorelbine, paclitaxel, cabazitaxel, and amrubicin.^9^, ^10^, ^11^ None of these studies provided any benefit.

Anthracycline‐based therapy could represent a relevant choice in this indication.^12^, ^13^ A phase 2 study evaluating second‐line doxorubicin–paclitaxel association in 46 ES‐SCLC patients highlighted interesting results with a response rate of 41%, including three complete responses (CR), a median PFS and OS of 14 and 25 weeks, respectively, suggesting a synergistic effect, with an acceptable safety profile.^14^ Grade 4 neutropenia occurred in 63% of cases (optional Granulocyte Colony‐Stimulating Factor [G‐CSF]). Anthracycline‐related cardiac failure could represent the main limited toxicity. Nevertheless, Torti et al. suggested a greater cardiac tolerance with epirubicin compared with doxorubicin.^15^ Two phase II studies highlighted the activity of epirubicin in SCLC both as monotherapy or when combined with other drugs.^16^, ^17^ Recently, Pasello et al. reported retrospective data of 68 patients treated with epirubicin at 70 mg/m^2^ combined with paclitaxel at 135 mg/m^2^ in the second line^18^ and highlighted the efficacy of this association. Partial response (PR) and stable disease (SD) were obtained in 30% and 34% of cases, respectively, with a median PFS and OS reaching 21.8 and 26.5 weeks, respectively. Nonetheless, this doublet is not standard of care for ES‐SCLC patients but remains an interesting therapeutic option in daily practice, especially in case of failure of approved therapies.

Unfortunately, real‐life data are lacking in the literature concerning the efficacy of this doublet and particularly for patients with central nervous system metastases. In this real‐life study, we evaluated the efficacy and safety of epirubicin and paclitaxel combination in a retrospective cohort of ES‐SCLC patients treated as the second line, with focused attention on BMs.

## 2 MATERIALS AND METHODS

### 2.1 Study population and procedures

EpiTax is a retrospective multicentric review of all consecutive patients with relapse SCLC and treated with an epirubicin–paclitaxel regimen after at least one line of platinum‐based chemotherapy between January 2010 and December 2020 in three French centers. Eligible patients were adults (age ≥ 18 years) with histologically or cytologically proven SCLC and who received at least one cycle of intravenous epirubicin at 90 mg/m^2^ on day 1 combined with paclitaxel at 175 mg/m^2^ on day 1 administered every 3 weeks until disease progression according to Response Evaluation Criteria in Solid Tumors (RECIST 1.1)^19^ or unacceptable toxicity according to the Common Toxicity Criteria for Adverse Events (CTCAE) version 4.0.^20^ The dosage was determined according to previous phase II studies in SCLC.^14^, ^17^ Patients with a history of BMs were included. Exclusion criteria were written or oral refusal to participate expressed during the lifetime, composite tumor or another subtype of lung carcinoma, and SCLC patients not treated with the study regimen.

Data concerning clinical and biological characteristics of patients, tumor profile, and successive local and systemic treatments were collected. Patients with tumor response to first‐line platinum were defined as sensitive when disease relapse or progression has been observed at least 90 days after the last administration of platinum‐based chemotherapy. Radiological assessment was performed approximately every 2 months (CT scan, MRI, and/or ^18^F‐FDG PET/CT).

### 2.2 Outcomes

The primary endpoint was PFS, defined as the time from epirubicin–paclitaxel regimen initiation to progressive disease (PD) or death from any cause. The secondary endpoints were OS (time from the date of the first infusion to death or loss to follow up), ORR (CR or PR) according to RECIST 1.1, duration of response (DOR) (time from first response to tumor progression or death from any cause), disease control rate (DCR), intracranial control rate (ICR), and safety according to the CTCAE version 4.0.

### 2.3 Statistical analysis

Efficacy and safety were assessed in all included patients who received at least one infusion of epirubicin combined with paclitaxel. Continuous variables were described by the number of no missing data, median, minimum, and maximum. Categorical variables were described as the total number and percentage per category. PFS, OS curves, and related 95% confidence intervals (CIs) were calculated with a survival analysis using the Kaplan–Meier method.

### 2.4 Study oversight

This noninterventional study was approved by a regional ethics committee and France's national data protection authority (CNIL), according to French law. All patients alive provided written informed consent before enrollment. The opposition should be expressed within 15 days in case of refusal.

## 3 RESULTS

### 3.1 Baseline characteristics

Between 2010 and 2020, a total of 29 patients were included with no screen failure reported. At the initiation of epirubicin–paclitaxel regimen, the main characteristics of patients were a median age of 60 (45–77) years, 19 (65.5%) males, 28 (96.6%) former or current smokers, and 21 (72.4%) PS 0–1 (Table 1). Twenty‐five (86.2%) and 4 (13.8%) patients had the extensive and limited disease at diagnosis, respectively. Eighteen presented multiple metastatic sites. Fifteen (51.7%) patients had a history of BMs before epirubicin–paclitaxel regimen initiation. Among them, 11 were previously treated with radiotherapy (6 with whole‐brain radiation therapy [WBRT], 2 with stereotactic radiation therapy [SRT], 2 with successively SRT and WBRT, and 1 with prophylactic cranial irradiation [PCI]).

**TABLE 1:** Patients baseline characteristics

Patients characteristics	*N* = 29
Age, median (range) year	60 (45–77)
Age category, no. (%)	
<65 years	18 (62.1)
≥65–75 years	10 (34.5)
≥75 years	1 (3.4)
Sex, no. (%)	
Male	19 (65.5)
Female	10 (34.5)
Smoking status, no. (%)	
Never smoker	1 (3.4)
Former smoker	10 (34.5)
Current smoker	18 (62.1)
First‐line treatment, no. (%)	
Platinum‐etoposide	26 (89.7)
Chemoimmunotherapy	3 (10.3)
Line of study regimen initiation	
Median (range)	3 (2–5)
2	11 (37.9)
3	11 (37.9)
4	6 (20.7)
5	1 (3.4)
ECOG PS, no. (%)	
0	3 (10.3)
1	18 (62.1)
≥2	6 (20.7)
MD	2 (6.9)
Best response to prior treatment, no. (%)	
Complete response	—
Partial response	12 (41.4)
Stable disease	5 (17.2)
Progressive disease	12 (41.4)
Not evaluated	—
Number of metastatic sites, no. (%)	
Single site	—
Multisites	29 (100)
History of brain metastases, no. (%)	15 (51.7)
Prior cerebral local treatment, no. (%)	
WBRT 30 Gy	6 (40)
SRT	2 (13.3)
SRT and WBRT	2 (13.3)
PCI 25 Gy	1 (6.7)
No treatment	4 (26.7)

Abbreviations: ECOG PS, Eastern Cooperative Oncology Group Performance Status; MD, missing data; PCI, prophylactic cerebral irradiation; SRT, stereotactic radiation therapy; WBRT, whole‐brain radiation therapy.

The median of previous systemic therapy lines was 2 (1–4). Eleven (37.9%) patients were treated with epirubicin–paclitaxel in the second line, whereas 11 (37.9%) received topotecan and 7 (24.1%) were rechallenged with platinum‐based doublet in this setting. The median treatment‐free interval before epirubicin–paclitaxel initiation was 2.8 months.

### 3.2 Efficacy

#### 3.2.1 Survival

**FIGURE 1:**
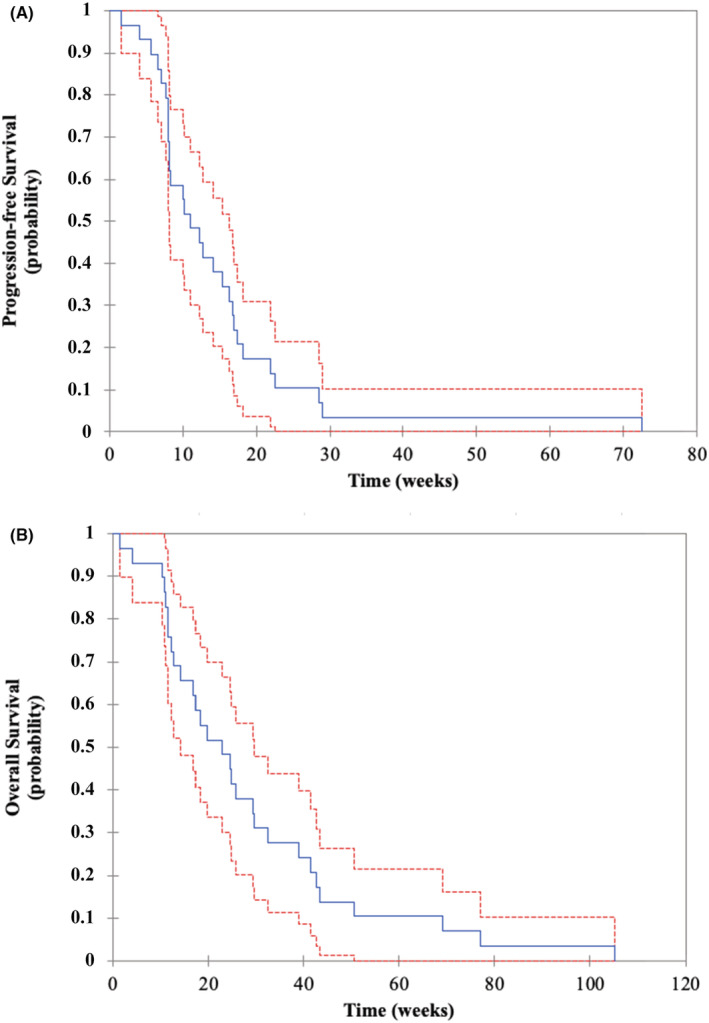
Kaplan–Meier estimates of (A) progression‐free survival and (B) overall survival. (A) The blue line represents the Kaplan–Meier curve for progression‐free survival, and the red lines represent the 95% confidence interval. (B) The blue line represents the Kaplan–Meier curve for overall survival, and the red lines represent the 95% confidence interval.

Time from initiation of the first‐line systemic treatment to death from any cause achieved 14.0 months (95% CI, 11.0–24.0). Median PFS and OS achieved 11.0 (95% CI, 8.1–16.3) and 23.0 (95% CI, 14.1–29.6) weeks, respectively (Figure 1A,B). All patients were dead at the end of the study.

#### 3.2.2 Response

ORR and DCR were 34.5% and 55.2%, respectively: 10 patients with PR and 6 with SD. Median DOR was 8.3 (95% CI, 8.0–8.9) weeks. Ten (34.5%) patients experienced PD at first evaluation (Table 2). Concerning the 15 patients with a history of BMs, ICR achieved 20% (3 patients). Cerebral progression concerned 10 (66.7%) of the 15 patients (Table 2). De novo brain metastases concerned 4 patients.

**TABLE 2:** Systemic and cerebral efficacy (intention to treat)

Efficacy of epirubicin–paclitaxel regimen	*N* = 29
Best overall response, no. (%)	
Partial response	10 (34.5)
Stable disease	6 (20.7)
Progressive disease	10 (34.5)
Not evaluated	3 (10.3)
Objective response rate, %	34.5
Disease control rate, %	55.2
History of BMs, no. (%)	(*N* = 15)
ICR	3 (20)
Progressive disease	10 (66.7)
Not evaluated	2 (13.3)

Abbreviations: BMs, brain metastases; ICR, intracranial control rate.

### 3.3 Subset analysis

#### 3.3.1 Impact of treatment lines

Eleven patients were treated with epirubicin–paclitaxel doublet in second line (Table 3). In this subgroup, median PFS and OS achieved 12.7 (95% CI, 5.6–16.7) and 14.1 (95% CI, 11.0–24.9) weeks, respectively. ORR and DCR were 45.5% and 63.6% (5 patients with PR and 2 with SD). PD concerned 1 (9.4%) patient. Eighteen patients were treated with epirubicin–paclitaxel doublet as third‐line treatment and beyond. Survival results and efficacy are reported in Table 3.

**TABLE 3:** Subset analysis

	Epirubicin–paclitaxel as second‐line treatment (*N* = 11)	Epirubicin–paclitaxel as third‐line treatment and beyond (*N* = 18)	Platinum‐resistant (*N* = 16)	Platinum‐sensitive (*N* = 13)
Best overall response, no. (%)				
Partial response	5 (45.4)	5 (27.8)	7 (43.7)	3 (23.1)
Stable disease	2 (18.2)	4 (22.2)	2 (12.5)	4 (30.8)
Progressive disease	1 (9.1)	9 (50)	5 (31.3)	5 (38.4)
Not evaluated	3 (27.3)	0 (0)	2 (12.5)	1 (7.7)
Objective response rate, %	45.4	27.8	43.7	23.1
Disease control rate, %	63.6	50	56.2	53.9
PFS, median (95% CI) (weeks)	12.7 (5.6–16.7)	9.1 (8.0–16.9)	12.5 (8.0–16.7)	8.3 (8.0–16.9)
OS, median (95% CI) (weeks)	14.1 (11.0–24.9)	27.6 (18.4–41.4)	21.5 (12.7–32.4)	23.0 (11.6–39.0)

Abbreviations: OS, overall survival; PFS, progression‐free survival.

#### 3.3.2 Impact of platinum‐sensitivity status

Thirteen (44.8%) and 16 (55.2%) patients were, respectively, considered as platinum‐sensitive and platinum‐resistant. Concerning platinum‐resistant patients, 10 received epirubicin–paclitaxel in the second line and six in the third line with median PFS and OS achieving 12.5 (95% CI, 8.0–16.7) and 21.5 (95% CI, 12.7–32.4) weeks, respectively. ORR and DCR were 43.8% (7 patients with PR) and 56.3% (2 patients with SD); PD was 31.3% (5 patients). Details are reported in Table 3.

### 3.4 Safety

All 29 patients were evaluable for safety (Table 4). The most common all‐grade nonhematological adverse events (AEs) observed were neurotoxicity (31%), asthenia (75.9%), anorexia (37.9%), stomatitis (13.8%), and nausea (24.1%). Concerning hematological AE, grade 1–2 neutropenia, anemia, and thrombocytopenia occurred in 10.3%, 55.2%, and 24.1%, respectively. Grade 3–4 AE concerned 7 patients: neutropenia (17.2%), asthenia (3.4%), anorexia (3.4%), anemia (3.4%), thrombocytopenia (3.4%), and nausea (3.4%). No febrile neutropenia or toxic death was reported. Prophylactic G‐CSF was used in 27 (93%) of patients.

**TABLE 4:** Most common treatment‐related adverse events (AEs) according to Common Toxicity Criteria for Adverse Events (*N* = 29)

	Grade 1–2, no (%)	Grade 3–4, no (%)
Hematological AE		
Anemia	16 (55.2)	1 (3.4)
Thrombocytopenia	7 (24.1)	1 (3.4)
Neutropenia	3 (10.3)	5 (17.2)
Nonhematological AE		
Asthenia	22 (75.9)	1 (3.4)
Anorexia	11 (37.9)	1 (3.4)
Neurotoxicity	9 (31.0)	—
Stomatitis	4 (13.8)	—
Cardiac failure	1 (3.4)	—
Nail toxicity	2 (6.8)	—
Hepatic cytolysis	1 (3.4)	—
Skin rash	1 (3.4)	—
Arthralgia	3 (10.3)	—
Nausea	7 (24.1)	1 (3.4)
Vomiting	1 (3.4)	—
Diarrhea	1 (3.4)	—

Abbreviation: AE, adverse events.

## 4 DISCUSSION

This multicenter real‐life study highlights the efficacy of epirubicin–paclitaxel doublet in patients with relapsed SCLC previously treated with a platinum‐based regimen. Indeed, we observed interesting results with median PFS and OS of 11.0 (95% CI, 8.1–16.3) and 23.0 (95% CI, 14.1–29.6) weeks and an ORR achieving 34.5%. The safety profile was acceptable.

These data are consistent with results reported by Pasello et al. who described a cohort of 68 patients treated with the same chemotherapy doublet in second‐line treatment.^18^ Status concerning BMs was not mentioned. The study reported an ORR and a DCR of 30% and 64%, a median OS and PFS of 26.5 and 21.8 weeks, respectively. In the same way, the EpiTax study confirms a clinical benefit of this regimen even across a second‐line treatment. Nevertheless, efficacy seemed to be more important in the Italian study, whereas epirubicin and paclitaxel were administered every 3 weeks at a dose of 70 and 135 mg/m^2^, respectively, versus 90 and 175 mg/m^2^, respectively, in our cohort. The median line of doublet initiation was 3 in our study which could explain a decreased efficacy. Toxicity was also quite similar. Indeed, the hematological toxicity profile by cycle had shown grade 3–4 neutropenia in 32% and grade 3–4 anemia in 2% of cases, without systematic use of G‐CSF. Other grade 3 nonhematological AE included fatigue (5%), nausea (2%), and vomiting (2%).

Topotecan remains currently the dominant second‐line therapy in ES‐SCLC with the results of a study comparing this chemotherapy versus best supportive care that showed an OS of 25.9 weeks versus 13.9 weeks, respectively, and a DCR of 51% (7% of PR and 44% of SD).^8^ Recent studies evaluating second‐line topotecan confirmed a median PFS and OS of 13 and 29 weeks, respectively.^6^, ^9^, ^10^, ^11^ In our study, we observed a trend of improved efficacy with epirubicin–paclitaxel doublet in the second line compared to topotecan with an ORR and a DCR achieving 45.5% and 63.6% versus 18.2% and 36.4%, respectively. However, these results should be interpreted with caution due to a small cohort of patients and the absence of direct comparison imposed by the study design. Moreover, other studies confirmed the unfavorable safety profile of topotecan with dramatic hematological toxicities in the foreground (until 54%, 31%, and 54% of grade 3–4 thrombocytopenia, anemia, and neutropenia, respectively, despite G‐CSF),^6^, ^9^, ^10^, ^11^ which could represent a serious limitation for its use in a pretreated symptomatic population in real life. On the other hand, the epirubicin–paclitaxel combination seemed to have a better hematological toxicity profile compared with topotecan. Finally, considering the regular cycle prescription of oral topotecan that systematically requires medical approval every 3 weeks, no hypothetic benefit concerning the quality of life appears compared to intravenous chemotherapy administered 1 day every 3 weeks.

In a phase 2 study, Trigo et al. highlighted the efficacy of lurbinectedin, an RNA polymerase II inhibitor, administered at 3.2 mg/m^2^ intravenously every 3 weeks in 37 patients.^21^ Median PFS and OS achieved 3.9 and 9.3 months. Side effects mainly consisted of hematological AE (grade 3–4 anemia 9%, thrombocytopenia 7%, leucopenia 29%, neutropenia 46%). Febrile neutropenia was notably reported in 10% of cases. In addition, nausea, vomiting, diarrhea, and fatigue represented the main nonhematological AE without any grade 3–4 reported. Nevertheless, in phase 3 ATLANTIS trial,^22^ lurbinectedin–doxorubicin association compared with the investigator's choice treatment (topotecan or CAV protocol) failed to meet its primary endpoint of OS, which raises questions about the place of lurbinectedin in the second‐line therapeutic strategy.

To our knowledge, the intracranial efficacy of epirubicin–paclitaxel doublet or paclitaxel monotherapy in SCLC is yet unclear, and no data are available in the literature. A Japanese study reported a benefit of amrubicin monotherapy in a small cohort (8 patients) of SCLC patients with BMs.^23^ ORR was 50% and the median time to progression (TTP) achieved 150.5 days. As a comparison, Korfel et al. highlighted the intracranial efficacy of topotecan in 30 patients previously treated with one (14) or two (16) lines of platinum‐based regimen.^24^ Eight patients received prior whole‐brain irradiation including 7 patients treated with PCI. Authors reported a cerebral ORR of 33% (including 10% of CR and 23% of PR), a DCR of 60%, and a median TTP of 3.1 months. However, the small sample size and nonuniform patient populations limited the interpretation of the results of these two studies. In EpiTax study, ICR achieved only 20%. Several mechanisms that may decrease blood–brain barrier penetration of chemotherapy are reported in the literature. Indeed, multidrug resistance proteins such as P‐glycoprotein (p‐gp), expressed by brain endothelial cells, may have a key role in cerebral resistance to cytotoxic in SCLC and other tumor types.^25^ It is possible that p‐gp, acting as an efflux pump, may have blocked both anthracycline and paclitaxel in our cohort. Targeting this transporter could constitute a promising area of research.

Relapsed SCLC within 6 weeks of completing first‐line chemotherapy and baseline PS ≥2 are mostly associated with lower response rates to further chemotherapy and shorter survival. In our study, however, PFS and ORR were superior in the platinum‐resistant subgroup treated with epirubicin–paclitaxel compared with platinum‐sensitive. It could be explained by the earlier initiation of the antracycline‐taxane regimen in this subgroup because of its platinum‐based rechallenge ineligibility. Indeed, we observed improvement of both ORR (45.4% vs. 27,8%) and DCR (64% vs. 50%) for patients treated with epirubicin–paclitaxel in the second line compared with those treated in the third line and after. However, this trend has not been confirmed regarding the survival results probably due to our small number of patients.

The limitations of our study mainly consisted of its retrospective design and a small cohort of 29 patients. However, the focused attention on cerebral efficacy in multicentric real‐life conditions constituted the strength of EpiTax. Based on these results, a randomized clinical trial would be justified to precise the place of this doublet.

## 5 CONCLUSION

Epirubicin–paclitaxel association highlighted efficacy for relapsed SCLC patients with interesting PFS and OS of 11 and 23 weeks, respectively, ORR of 34.5%, and a tolerable safety profile in real‐life conditions. This doublet could represent another valuable therapeutic option in this indication. These results would deserve further investigations in a randomized prospective cohort.

## AUTHOR CONTRIBUTION

Conception and design of the study: J. Annic, M. Geier, and G. Robinet. Acquisition of data: J. Annic, H. Babey, R. Corre, and V. Bourbonne. Analysis and/or interpretation of data: J. Annic, M. Geier, G. Robinet, R. Descourt, and G. Quéré. Drafting the manuscript: J. Annic, M. Geier, G. Robinet, H. Babey, R. Corre, and G. Quéré. Revising the manuscript critically for important intellectual content: All authors. Approval of the version of the manuscript to be published (the names of all authors must be listed): J. Annic, M. Geier, H. Babey, R. Corre, R. Descourt, G. Quéré, E. Renaud, M. Lambert, P. Le Noac' h, E. Dhamelincourt, J. Nguyen, A. Vu, V. Bourbonne, G. Robinet.

## FUNDING INFORMATION

The authors received no financial support for the research, authorship, and/or publication of this article.

## CONFLICT OF INTEREST

The authors confirm that there is no known conflict of interest associated with this publication and there has been no significant financial support for this work that could have influenced its outcome.

## ETHICS STATEMENT

This noninterventional study was approved by a regional ethics committee and France's national data protection authority (CNIL), according to French law.

## Data Availability

This non‐interventional study was approved by a regional ethics committee and France's national data protection authority (CNIL), according to French law.
